# The circuit basis for chronic pain and its comorbidities

**DOI:** 10.1097/SPC.0000000000000650

**Published:** 2023-04-25

**Authors:** Ryan Patel

**Affiliations:** King’s College London, Wolfson Centre for Age Related Diseases, Guy’s Campus, Newcomen Street, London, UK

**Keywords:** chronic pain, comorbid mood disorders, neural circuits

## Abstract

Chronic pain is poorly treated with many developing disabling comorbidities such as anxiety, depression and insomnia. Considerable evidence supports the idea that pain and anxiodepressive disorders share a common neurobiology and can mutually reinforce, which has significant long-term implications as the development of comorbidities leads to poorer treatment outcomes for both pain and mood disorders. This article will review recent advances in the understanding of the circuit basis for comorbidities in chronic pain.

**Recent findings:**

A growing number of studies have aimed to determine the mechanisms underlying chronic pain and comorbid mood disorders by using modern viral tracing tools for precise circuit manipulation with optogenetics and chemogenetics. These have revealed critical ascending and descending circuits, which advance the understanding of the interconnected pathways that modulate the sensory dimension of pain and the long-term emotional consequences of chronic pain.

**Summary:**

Comorbid pain and mood disorders can produce circuit-specific maladaptive plasticity; however, several translational issues require addressing to maximise future therapeutic potential. These include the validity of preclinical models, the translatability of endpoints and expanding analysis to the molecular and system levels.

## INTRODUCTION

Effective treatment of chronic pain is a major unmet clinical challenge, leading to long-term disability and substantial socioeconomic loss. Although estimates vary, the prevalence of chronic pain amongst the adult population in Europe and the USA is thought to be 19% ^1^,^2▪▪^, over half of whom have suffered for more than 2 years, and 40% of whom report inadequate pain relief from currently available analgesics ^1^. The prevalence of mild-to-severe depression more than doubles in those living with chronic pain (8.7–22.3%, respectively) compared with those without (1.2–8.8%) ^2▪▪^, and these figures may rise considerably in fibromyalgia ^3^. Long before the formal prescribing of antidepressant treatments for chronic pain, the concept of a link between anxiodepressive disorders and chronic pain was obvious, and the possibility of a common neurobiology encouraged the use of antidepressants for those that experienced both pain and depression. The logic was that improving mood and wellbeing would lessen the burden of persistent pain. It is now understood that pain relief from antidepressants can be independent of any actions on mood ^4^. Moreover, it is recognised that chronic pain and comorbidities can mutually reinforce, and the combination can be more disabling than either condition alone, leading to poorer treatment outcomes for pain and depression ^5^. Understanding their reciprocal interaction at a circuitry level is a vital first step towards optimal therapeutic targeting.

**KEY POINTS**

- Chronic pain is frequently associated with mood disorders which can mutually reinforce.
- The presence of co-morbidities can lead to poorer treatment outcomes.
- Adaptations within discrete circuits underlie changes in sensory and affective processing in chronic pain states.

## ASSESSING THE EMOTIONAL AND COGNITIVE CONSEQUENCES OF CHRONIC PAIN IN PRECLINICAL MODELS

The translatability of preclinical pain and mental health research has been a contentious issue for a number of years now. Many of the emotional consequences of chronic pain can be modelled in rodents, which tend to emerge after 4 weeks postinjury, but to identify the circuit basis for these comorbidities, valid measures are required. Clearly, the full spectrum of symptoms experienced during depression cannot be replicated in rodents, and more appropriate terminology is required to describe animal behaviour; however, more recently, many behavioural assays have been refined to assess affective state-related behaviour in a manner that may better relate to particular aspects of anxiodepressive disorders. As a prey species, studying anxiety-like behaviours in rodents is still heavily reliant on their hesitance to explore novel, brightly lit, or open environments. Advances in home cage monitoring allow for studying open-field behaviour, where animals can be studied during their active phase without additional confounding stressors such as unfamiliar environments and the presence of the experimenter ^6^. Other ethological endpoints include social interactions and nesting but also motivation to perform homoeostatic behaviours such as feeding and grooming ^7^. The sucrose preference test is most frequently used to assess anhedonia, but the reward learning assay permits more nuanced study of reward sensitivity, and an analogous human test also exists ^8^. Cognitive impairments, including attention, executive functioning and working memory, are commonplace in chronic pain and major depressive disorder ^9^; these are frequently studied preclinically using assays, including novel object recognition and operant learning. Ideally, studies should utilise a range of tests to link precise circuit function to diverse behaviours.

## NEURAL CIRCUITS FOR PAIN AND AFFECTIVE PROCESSING

### Lateral habenula

Considering its role in regulating negatively motivated behaviour, the lateral habenula, located in the epithalamus, has attracted much attention as a pharmacotherapeutic target for mood disorders over the last decade. Afferent projections arise from diverse limbic structures and the basal ganglia, and through direct and indirect innervations, the lateral habenula can engage serotonergic, noradrenergic and dopaminergic systems. For these reasons, this nucleus is uniquely placed to act as an integrative hub for value-based, sensory and experience-dependent information, capable of controlling a wide range of motivational, behavioural and cognitive processes ^10^. As part of its function in encoding aversive states, the lateral habenula afferent and efferent pathways are implicated in pain and analgesia. Single unit recordings in rats revealed neuronal populations excited or inhibited by noxious somatosensory stimulation ^11^, and nerve injury resulted in higher levels of spontaneous or burst firing ^12,13^. Electrical stimulation of the lateral habenula produced an analgesic effect during acute inflammation ^14^.

An elegant study by Zhou *et al*. ^15^ described a trisynaptic circuit that mediated anxiety-like behaviours in a chronic pain state. Using viral tracing methods, this revealed a subpopulation of serotonergic dorsal raphe neurones projecting to somatostatin positive neurones in the central amygdala (CeA^Som^). Reduced serotonergic drive decreased the excitability of CeA^Som^ neurones in a 5-HT_1A_R-dependent manner. Optogenetically activating this pathway reversed anhedonia and learned helplessness behaviours after nerve injury. These excitatory CeA^Som^ neurones were subsequently shown to project to and excite glutamatergic neurones in the lateral habenula, which were hyperexcitable after nerve injury, and optogenetically inhibiting this pathway attenuated the same behaviours. Most CeA neurones are GABAergic, and inhibitory CeA^Som^ and CeA^PKCδ^ neurones represent two largely nonoverlapping populations with opposing antinociceptive and pronociceptive functions, respectively ^16^ suggesting that within the CeA discrete circuits drive sensory and affective adaptations in chronic pain states. This is also evident within the lateral habenula. The NK3R was found to be downregulated in the lateral habenula following trigeminal nerve ligation. Unilateral (ipsilateral to the injury) chemogenetic inhibition of, or NK3R overexpression in, excitatory lateral habenula neurones reversed anxiety-like behaviour but not mechanical hypersensitivity following nerve injury, whereas bilateral silencing reversed both behaviours ^12^. Furthermore, optogenetically activating an excitatory neurokinin positive projection from the periaqueductal grey to the lateral habenula reduced the frequency of excitatory postsynaptic currents in habenula neurones, and chemogenetic silencing this pathway prevented the antinociceptive and anxiolytic effect of NK3R overexpression in the lateral habenula ^17^.

The lateral habenula may also be a key locus for opioid analgesia. In naive animals, activation of μ-opioid receptors decreased neuronal activity in the lateral habenula via postsynaptic hyperpolarisation and presynaptic inhibition of glutamate release ^18^. Microinjection of μ-opioid receptor agonists herein increased paw withdrawal thresholds after nerve injury and produced conditioned place preference, an indirect readout of a positive affective state ^19^. As the former represents a spinally mediated sensorimotor response, it would suggest that the actions of opioid receptor agonists within the lateral habenula are at least partly through subsequent engagement of the descending pain modulatory system. Within the ascending circuits, optogenetically activating a lateral preoptic hypothalamic to habenula projection produced place aversion that was reversed by μ-opioid receptor activation in the lateral habenula ^19^.

### Locus coeruleus

Historically, the locus coeruleus was considered homogenous both anatomically and functionally. The advent of modern tracing techniques has challenged this view, proposing a modular organisation of the locus coeruleus, which forms functionally segregated domains with respect to their efferent projections. Retrograde labelling from the spinal cord revealed a population of neurones located within the ventral aspect of the locus coeruleus and chemogenetic activation of this module supressed spinal neuronal excitability ^20^. After a nerve injury, an asymmetry developed in mediating descending pain modulation and affective behaviours. Within the ipsilateral descending pathway, in the early response to nerve injury, descending inhibitory function increased before extinction. In the later stages, where the emotional consequences of persistent pain become more evident, silencing either the ipsilateral or contralateral nucleus reversed learned helplessness behaviours ^21▪▪^. Dependent on the precise aetiology and time-dependent plasticity, the locus coeruleus can switch from ‘pain inhibitor’ to ‘pain generator’. Within the descending pathways, chemogenetic silencing of a locus coeruleus to dorsal reticular nucleus projection was antinociceptive in a model of neuropathic pain ^22^ mediated via α_1_-adrenoreptors in the dorsal reticular nucleus ^23^. As further evidence for the existence of parallel and functionally discrete noradrenergic pathways, optogenetic activation of the coeruleospinal module abolished the expression of diffuse noxious inhibitory controls (DNIC) ^24^. DNIC represent a unique form of endogenous modulation recruited by two spatially distinct heterosegmental noxious stimuli and are considered to underpin conditioned pain modulation. It is known that DNIC are subserved by noradrenergic signalling ^25^, but these inhibitory controls are expressed via the A5 nucleus as the final brainstem relay ^26▪^. Although both DNIC and the locus coeruleus are historically viewed as exerting inhibitory control over the spinal cord, this interaction implies that hyperactivity within the locus coeruleus could indirectly abolish the expression of parallel inhibitory pathways.

A growing body of evidence supports a functional dichotomy between the ascending and descending locus coeruleus modules. Neurones within the locus coeruleus with ascending projections reside in the dorsal aspect of the nucleus and are largely nonoverlapping with the coeruleospinal module. Chemogenetic activation of the locus coeruleus to prelimbic cortex pathway was aversive and anxiogenic in neuropathic rats but had no effect on nociceptive thresholds ^20^. Within the reciprocal pathway, the prelimbic to locus coeruleus projection underlies sex differences in cognitive deficits during inflammatory pain ^27^. Alongside the prelimbic cortex, the anterior cingulate cortex is associated with signalling the aversive dimension of pain but also more generally negative emotional experiences. Bilateral chemogenetic silencing of the locus coeruleus to anterior cingulate cortex projection had no effect on nociceptive thresholds in neuropathic rats but reversed despair-like behaviour ^21▪▪^. In addition to these direct cortical projections, engagement of limbic circuitry can also determine affective behaviours. In the absence of injury or an external stressor, optogenetic, or chemogenetic stimulation of the locus coeruleus to the basolateral amygdala pathway increased basal neuronal activity in the amygdala and produced conditioned place aversion and anxiety-like behaviours in a β-adrenoceptor dependent manner ^28,29^. Conversely, after nerve injury, silencing this pathway reversed open-field avoidance and conditioned fear learning without affecting nociceptive thresholds ^28^. Collectively, these data support the that modulation of nociceptive, cognitive and anxiogenic behaviours are separable functions within the locus coeruleus.

### Lateral hypothalamus

The lateral hypothalamus is functionally and molecularly diverse, with a critical role in coordinating diverse physiological and behavioural functions including arousal, sleep-wake states, feeding and stress ^30^. Both the lateral septum and bed nucleus of the stria terminalis (BNST) act as relays to integrate contextual inputs and can engage discrete circuits within the lateral hypothalamus. The lateral septum integrates cortical information about the social environment and prior experiences into downstream executive regions ^31^. The BNST is implicated in mediating overlapping behaviours including social attachment, offspring bonding, stress and sustained fear states ^32^. In a model of inflammation with comorbid anxiety and chronic stress with comorbid mechanical hypersensitivity, neuronal activity in the lateral septum was increased compared with naive mice when animals were exploring open arm areas versus the closed arms areas in the plus maze test ^33▪^. A reduction in nociceptive withdrawal threshold and anxiety-like behaviours could be reproduced in naive mice through optogenetic activation of these GABAergic lateral septum neurones. Conversely, optogenetic or chemogenetic silencing of these neurones produced resilience to pain and anxiety in both models. Viral circuit tracing revealed lateral septum GABAergic neurones project to multiple downstream targets, including the nucleus accumbens, ventral tegmental area, periaqueductal grey, hippocampus and throughout the hypothalamus; these were largely nonoverlapping populations. In particular, activation of the lateral septum to lateral hypothalamus pathway was pronociceptive and anxiogenic in naive mice, and inflammation or chronic stress increased inhibitory synaptic input to the lateral hypothalamus from the lateral septum. Together, these results identified a convergent circuit for the interaction of comorbid pain and anxiety; however, parallel circuits may exist within the lateral hypothalamus with segregated functions. Likewise, neurones within the BNST are also largely GABAergic and provide inhibitory input to the lateral hypothalamus, but this circuit selectively regulated anxiety-like behaviours but not nociceptive thresholds ^34^. Chemogenetic silencing of the BNST to lateral hypothalamus pathway promoted anxiety-like behaviours in the open field, plus maze and light-dark tests in naive mice, and activating this circuit reversed chronic pain-induced anxiety. These effects were attributed to CART-positive interneurones, which mediate local inhibitory modulation within the BNST. Chronic pain led to intrinsic changes in the excitability of these interneurones, which in turn provided increased inhibitory inputs to lateral hypothalamus-projecting BNST neurones.

### Thalamocortical

In their study, Zhu *et al.* ^35▪^ studied two thalamocortical pathways and their role in mediating nociceptive hypersensitivity resulting from either nerve injury or chronic stress. The posterior thalamus and parafascicular nucleus relay sensory information to the cortex either via the spinothalamic tract or thalamo-thalamic signalling. After nerve injury or inflammation, posterior thalamic neurones, but not parafascicular neurones, were hyperexcitable to low-intensity mechanical stimuli. However, in a model of chronic stress, parafascicular neurones, but not posterior thalamic neurones, exhibited reduced excitability as shown previously to result from inhibition from the central nucleus of the amygdala ^36^. Precise chemogenetic silencing of the glutamatergic posterior nucleus to S1 pathway alleviated nerve injury-induced mechanical hypersensitivity. In the parafascicular to anterior cingulate cortex pathway, reduced feed-forward inhibition resulted in cortical neuronal hyperexcitability, which was reversed by chemogenetic activation of this circuit, as were mechanical hypersensitivity and anxiety-like behaviours ^35▪^. Nociceptive hypersensitivity as a result of either tissue injury or chronic stress produced circuit-specific maladaptation and may indicate how anxiety leads to increased pain sensitivity in chronic pain states.

## CONCLUSION

Perceived pain intensity is not always proportional to the extent of tissue or nerve damage but depends on a complex combination of physiological, cognitive, emotional and sociocultural factors and can be strongly influenced by comorbidities such as stress, anxiety, depression and sleep disturbances. The last 2 decades have delivered major advances in our understanding of the neurobiology of somatosensation within peripheral and spinal circuits, but the brain circuits that sustain chronic pain remain a more open question. When treating chronic pain, the goal of effective treatment is to improve quality of life while also achieving pain relief. When developing treatment algorithms, both sensory and affective factors should be taken into consideration as these may affect treatment choice and outcomes. Identifying the circuit basis for pain and its comorbidities represents the first step in identifying novel targets. Expanding analysis into the systems and transcriptional levels and refining preclinical models and endpoints will be central to maximising therapeutic potential.

Box 1. Key points

- Chronic pain is frequently associated with mood disorders, which can mutually reinforce each other.
- The presence of comorbidities can lead to poorer treatment outcomes.
- Adaptations within discrete circuits underlie changes in sensory and affective processing in chronic pain states.
